# Systematic Review and Meta-Analysis of Telecoaching for Self-Care Management among Persons with Type 2 Diabetes Mellitus

**DOI:** 10.3390/ijerph20010237

**Published:** 2022-12-23

**Authors:** Hesti Platini, Artanti Lathifah, Sidik Maulana, Faizal Musthofa, Shakira Amirah, Muhammad Fahd Abdurrahman, Maria Komariah, Tuti Pahria, Kusman Ibrahim, Juan Alessandro Jeremis Maruli Nura Lele

**Affiliations:** 1 Department of Medical Surgical Nursing, Faculty of Nursing, Universitas Padjadjaran, Bandung 45363, Indonesia; 2 Professional Nurse Program, Faculty of Nursing, Universitas Padjadjaran, Bandung 45363, Indonesia; 3 Faculty of Medicine, Universitas Indonesia, Depok City 16424, Indonesia; 4 Department of Fundamental Nursing, Faculty of Nursing, Universitas Padjadjaran, Bandung 45363, Indonesia; 5 Faculty of Medicine, Universitas Kristen Indonesia, Jakarta 13630, Indonesia

**Keywords:** COVID-19, clinical outcome, diabetes mellitus, self-care, telecoaching

## Abstract

Background: In response to the need for safe care for people with diabetes mellitus in the current outbreak of COVID-19, it is critical to evaluate the model, service delivery, feasibility, and efficiency of diabetes mellitus telecoaching. Objective: This study aimed to conduct a systematic review and meta-analysis of the model and efficacy of telecoaching to improve self-care and clinical outcomes. Methods: This study uses the Preferred Reporting Item for Systematic Review and Meta-Analysis (PRISMA). We searched on 22 March 2022, using keywords that matched the MeSH browser in four databases to find relevant studies, namely, PubMed/Medline, Proquest, Scopus, and EBSCOhost. Additionally, we collected randomized controlled trials (RCTs) on Google Scholar using the snowball technique. A quality assessment was performed using the Cochrane Collaboration’s Risk of Bias tool (RoB)2. The meta-analysis used the DerSimonian–Laird random-effects model to analyze the pooled mean difference (MD) and its *p*-value. Results: Thirteen RCT studies were included for the systematic review and meta-analysis with a total number of participants of 3300. The model of telecoaching is a form of using nurses-led telephone and mobile apps, which are relatively cost-effective. The meta-analysis showed a positively improved statistically significance in clinical outcomes, including in HbA1c (a pooled MD of −0.33; 95% CI: −0.51–−0.15; *p* = 0.0003), blood glucose (−18.99; 95% CI: −20.89–−17.09; *p* = 0.00001), systolic blood pressure (−2.66; 95% CI: −3.66–−1.66; *p* = 0.00001), body mass index (−0.79; 95% CI: −1.39–−0.18; *p* = 0.01), and weight (−2.16 kg; 95% CI: −3.95–−0.38; *p* = 0.02). It was not, however, statistically significant in diastolic blood pressure (−0.87; 95% CI: −2.02–0.28; *p* = 0.14), total cholesterol (−0.07; 95% CI: −0.26–0.12; *p* = 0.46), low-density lipoprotein (−2.19; 95% CI: −6.70–2.31; *p* = 0.34), triglycerides (−13.56; 95% CI: −40.46–13.35; *p* = 0.32) and high-density protein (0.40; 95% CI: −1.12–1.91; *p* = 0.61). Conclusions: The telecoaching with nurses-led telephone and mobile apps significantly affected clinical outcomes on HbA1c, systolic blood pressure, weight, and BMI. Moreover, there was no significant effect on the total cholesterol, low-density lipoprotein, triglycerides, and high-density lipoprotein. Thus, telecoaching has the potential as a care model in diabetes mellitus during COVID-19 and similar pandemics to improve self-care and clinical outcomes, but all the studies analyzed involved non-COVID-19 patients, limiting the generalizability of the results to COVID-19.

## 1. Introduction

Since the outbreak of the coronavirus disease 2019 (COVID-19) pandemic in 2019, the severe acute respiratory syndrome caused by coronavirus 2 (SARS-CoV-2) has now developed into a global health threat. It has been declared by the World Health Organization (WHO) to be a Public Health Emergency of International Concern (PHEIC) [1]. Patients with certain medical conditions, such as diabetes mellitus, are identified by the Centers for Disease Control and Prevention (CDC) as having a high probability of developing critically severe COVID-19 [2]. During the COVID-19 pandemic, people with diabetes mellitus spent more time indoors to protect themselves and currently, many people with diabetes mellitus choose to postpone or cancel their healthcare appointments. This can lead to reduced support that diabetic individuals need to manage their disease and an increased risk of developing complications [3].

In reducing and preventing complications in people with DM, it is necessary to increase good self-management. Self-management is an individual effort to manage the disease, symptom management, treatment, and lifestyle changes [4]. Diabetes self-management behaviors are needed to achieve an optimal glycemic control, reduce the risk of complications, and improve health outcomes [5]; therefore, appropriate home health technologies can be important in helping people with DM to develop the self-management skills necessary to live with their disease.

Telecoaching is a telehealth service that aims to help and encourage patients to achieve their desired goals, a better quality of life, and to solve the relevant and experienced problems [6]. Training programs or coaching through mobile applications are the only diabetes education programs digitally delivered and recognized by the American Diabetes Association [7].

Therefore, in response to the need for the safe care of a person with diabetes mellitus (PWD) in the present issue of COVID-19, it is important to assess the model, service delivery, feasibility, and efficiency of telecoaching for diabetes mellitus. A review of the various telecoaching models can assist developers in understanding the shortcomings of the existing telecommunications programs and they can help incorporate different creative features and technologies in developing future models. Nurses and other healthcare professionals can also analyze the various telecoaching models and propose the most efficient telecoaching model for patients, enabling them to control their health better.

In this regard, this review aims to explore the potential of telecoaching for self-management for people with diabetes mellitus during COVID-19 and similar pandemics.

## 2. Methods

### 2.1. Study Design

This study used a scoping review following the Preferred Reporting Items for Systematic Review and Meta-Analysis guidelines [8]. Telecoaching for self-care models related to diabetes mellitus can be thoroughly studied using the scope or sub-group method [9]. An initial broad research topic was narrowed down during the scoping review process to comprehensively evaluate the efficacy outcomes. We began by asking: Which models and types of care delivery services promote acceptance, efficacy, and efficiency for self-care?

### 2.2. Search Strategy

This study used four databases to search for relevant studies, namely, PubMed/Medline, Proquest, Scopus, and EBSCOhost. Additionally, we collected data on Google Scholar using the snowball technique. We searched on 22 March 2022, using keywords that matched the MeSH browser.

These included for PubMed and Proquest: “telecoaching”[All Fields] AND (“diabetes mellitus”[MeSH Terms] OR (“diabetes”[All Fields] AND “mellitus”[All Fields]) OR “diabetes mellitus”[All Fields]); for Scopus: ((telecoaching) OR (tele-coaching)) AND (diabetes mellitus); and for EBSCOhost: “telecoaching” AND (“diabetes type 2” or “diabetes mellitus type 2” or “diabetes 2”).

### 2.3. Eligibility Criteria

The inclusion criteria followed the PICO framework (patient/problem, intervention/exposure, comparison/control, outcome) and comprised (1) the type of study: RCTs; (2) the study population: people with diabetes mellitus; (3) the intervention: telecoaching; (4) the outcomes: HbA1c, total cholesterol, SBP, DBP, BMI, HDL, LDL, triglycerides, blood glucose, and weight using the respective tools in mean, standard deviation, and p-value for the pre- and post-intervention and control; and (5) other care as the control. Meanwhile, the exclusion criteria were set to (1) studies that were not complete at the time of retrieval; (2) studies with irretrievable full-text articles; and (3) studies in languages other than English as an international language. Furthermore, a duplicate removal was also performed using EndNote X9 software (Clarivate, Philadelphia, PA, USA). The titles and abstracts of studies were screened according to the criteria of accessibility by three independent reviewers (i.e., SM, SA, and MFA). Any disagreements were discussed to consensus.

### 2.4. Data Collection and Analysis

The collecting data in this study followed the PRISMA flow diagram, including the identification of studies in the databases; screening for duplicates, titles, and abstracts; assessing full eligibility text; and an extraction and analysis of the included studies. We extracted studies manually on the extraction tabulation. The item data on the extraction were, namely, the author, country, design study, characteristic and number of samples, model, feasibility, and efficacy of telecoaching. We also extracted outcomes including the HbA1c, total cholesterol, systolic blood pressure, diastolic blood pressure, BMI, HDL, LDL, triglycerides, blood glucose, and weight. Additionally, we analyzed this study using a quantitative and qualitative analysis.

### 2.5. Quantitative Data Analysis

A statistical analysis was performed using Review Manager ver. 5.4 (The Nordic Cochrane Center, The Cochrane Collaboration, Copenhagen, Denmark). The mean differences and standard deviations with a 95% confidence interval (CI) and the *p*-values were extracted from studies for both the pre- and post-intervention and intervention versus the control post-treatment. We then interpreted the pooled effects using random-effects models. The main results used in the statistical analysis were the mean difference between the pre- and post-treatment using telecoaching for people with diabetes mellitus, which was shown by decreasing levels in HBA1C, blood glucose, total cholesterol, triglycerides, LDL BMI, weight, blood pressure, and increasing HDL in the respective tools used, as well as the mean difference between the telecoaching and control groups. The mean difference with a 95% CI and its respective *p*-value was used to determine the efficacy of the telecoaching on all outcomes, which was presented in a forest plot. We used an inverse variance and DerSimonian–Laird random-effects model as proposed by Riley et al., as we considered that a heterogeneity outside the study could also be discovered. The heterogeneity was further evaluated using estimated effect (I^2^)statistics based on the Cochrane threshold, with cut-off limits of 0%, 25%, 50%, and 75% as being an insignificant, low, moderate, and high heterogeneity, respectively [10]. According to von Hippel, the I^2^ is substantial when the number of studies is small. Following Duval and Tweedie, we also performed a sensitivity analysis using a trim and fill [11]. The sensitivity test was performed using the Jamovi 2.2.5 software and used when the heterogeneity was high.

## 3. Results

### 3.1. Selection Study

**Figure 1:**
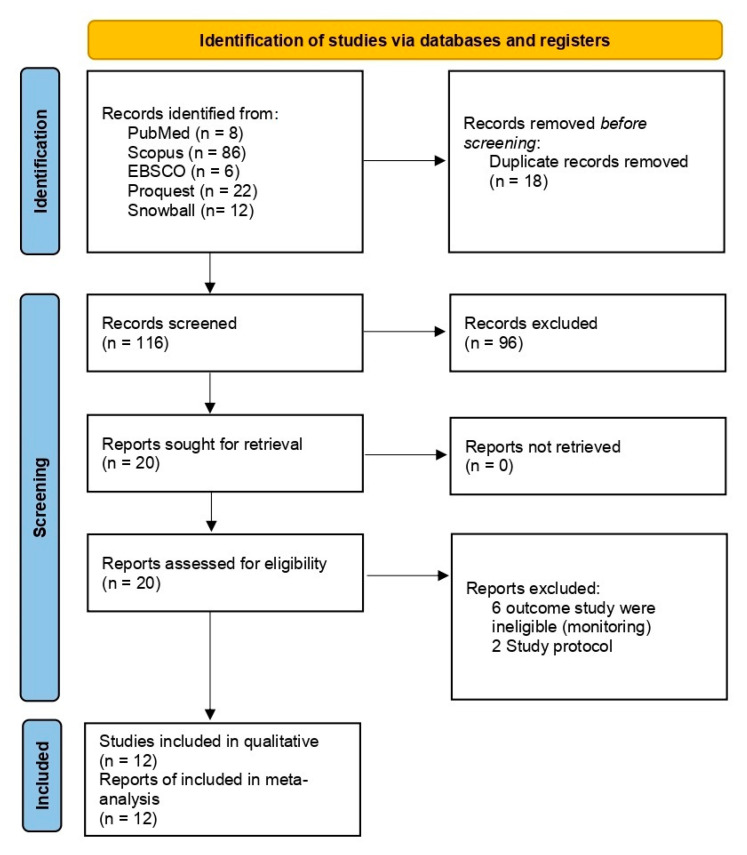
PRISMA flow diagram.

The process of conducting a literature search is depicted in the flowchart shown in Figure 1. A total of 126 studies were found due to thorough initial searches. After eliminating 18 duplicates, the authors screened the titles and abstracts, and 13 studies were retrieved for full-text evaluations after a thorough screening process [7,12,13,14,15,16,17,18,19,20,21,22,23]. We excluded four studies because their protocol studies and monitoring outcomes were ineligible for inclusion in the study. A total of thirteen studies were included in the qualitative analysis and quantitative analysis.

### 3.2. Characteristics of Included Studies

All the study designs used included a randomized controlled trial (RCT). The studies were conducted in the following locations: the United States of America (n = 4), Australia (n = 1), Belgium (n = 2), Canada (n = 1), Brazil (n = 1), Iran (n = 1), and South Korea (n = 2). People with Type 2 diabetes were among those who participated, with the average age of the respondents between the range of being early elderly and elderly [7,12,13,14,15,16,17,18,19,20,21,22,23]. The intervention group received telecoaching with the various models shown in Table 1. The control group received usual or routine care, which may have included a referral to face-to-face diabetes educators, nutritionists, and specialists. Additionally, in the intervention group, there was not only education focusing on therapy adherence and lifestyle, but people also received medication adjustments, exercise adjustments, and frequent blood glucose monitoring took place during these consultations in several studies [13,17,18,19,20].

### 3.3. Risk of Bias

**Figure 2:**
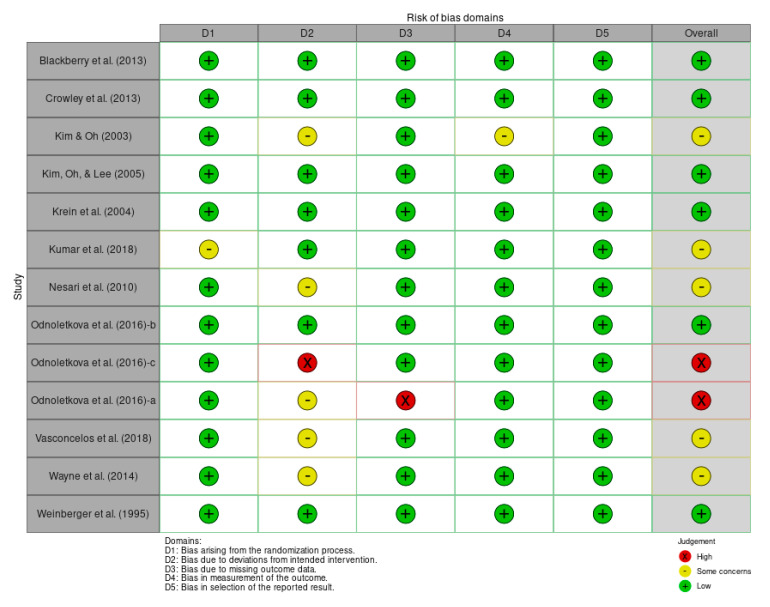
Traffic light plot’s risk of bias [7,12,13,14,15,16,17,18,19,20,21,22,23].

**Figure 3:**
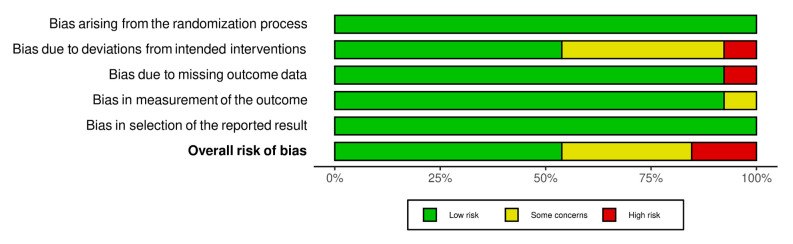
Summary risk of bias.

Overall, the included studies had a low risk of bias as individuals. Four studies were of some concern, and two were highly-biased studies (see Figure 2). The assessment component was highly biased, mostly in the bias due to a deviation from the intended deviation and missing outcome data. Additionally, with the component that was lowly-biased, the bias mostly arose from the randomization process and selection of the reported results (see Figure 3).

### 3.4. Study Outcome

#### 3.4.1. Model of Telecoaching

Most of the studies used a telecoaching model in the form of nurses-led telephone sessions. Meanwhile, two studies use an application as an intervention [7,14]. The telecoaching was carried out in the intervention and control groups using varying durations and frequencies as well as a follow-up.

#### 3.4.2. Meta-Analysis Outcome of HbA1c Reduction

**Figure 4:**
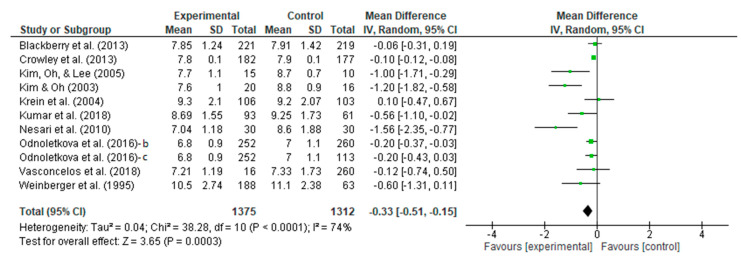
Forest plot intervention vs. control of telecoaching in achieving a HbA1C outcome [7,12,13,15,16,17,18,19,20,21,22].

**Figure 5:**
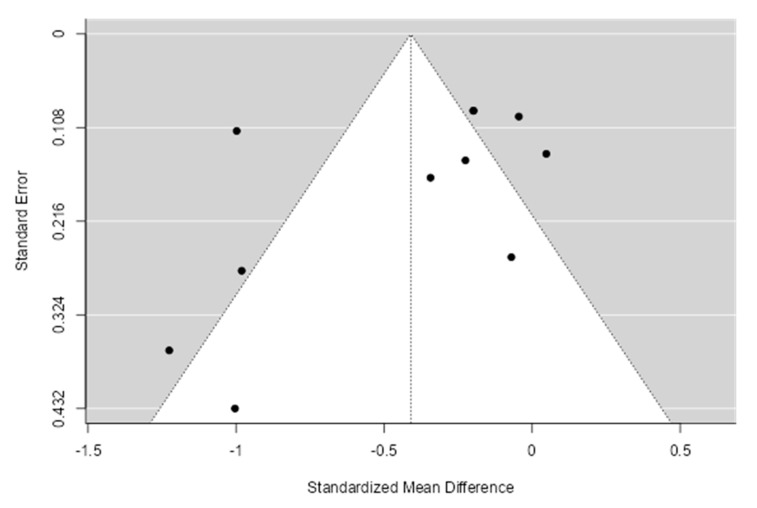
Funnel plot intervention vs. control of telecoaching in achieving a HbA1C outcome.

A meta-analysis assessed the intervention vs. control efficacy of the telecoaching in terms of HbA1c. The results shown in Figure 4 depict a significant effect (*p* = 0.0003) with a pooled MD of −0.33 (95% CI: −0.51–−0.15). The telecoaching was found to decrease Hb1Ac in people with DM significantly. A heterogeneity was also found (I^2^ = 74%; *p* < 0.0001). Additionally, sensitivity tests using the trim and fill method identified one study that was an outlier. Figure 5 depicts the distribution of the studies based on their mean difference and standard deviation.

#### 3.4.3. Meta-Analysis Outcome of Fasting Plasma Glucose Reduction

**Figure 6:**
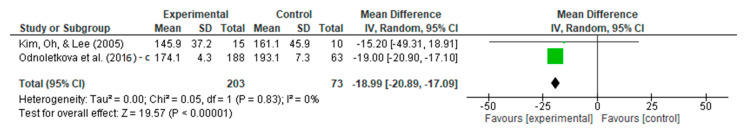
Forest plot intervention vs. control of telecoaching in achieving a blood glucose outcome [16,21].

A meta-analysis assessed the intervention vs. the control efficacy of telecoaching on blood glucose. The result is shown in Figure 6, which depicts a significant effect with a *p* < 0.00001 with a pooled MD of −18.99 (95% CI: −20.89–−17.09). The telecoaching was found to decrease the fasting plasma glucose in people with DM significantly. A heterogeneity was not found (I^2^ = 0%; *p* = 0.83).

#### 3.4.4. Meta-Analysis Outcome of Systolic Blood Pressure

**Figure 7:**
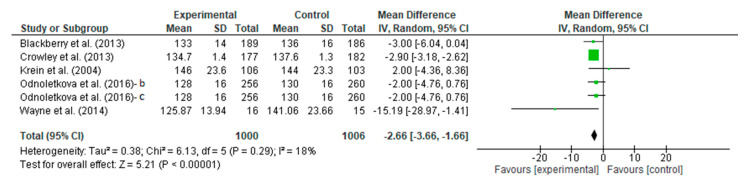
Forest plot intervention vs. control of telecoaching in achieving a SBP outcome [12,13,14,18,21,22].

A meta-analysis assessed the intervention vs. the control efficacy of telecoaching on the systolic blood pressure. The result is shown in Figure 7, which depicts no significant effect with a *p* < 0.00001 with a pooled MD of −2.66 (95% CI: −3.66–−1.66). The telecoaching was found to decrease the SBP in people with DM significantly. The heterogeneity was found to be low (I^2^ = 18%; *p* = 0.29).

#### 3.4.5. Meta-Analysis Outcome of Diastolic Blood Pressure

**Figure 8:**
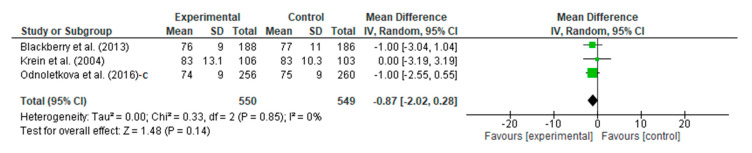
Forest plot intervention vs. control of telecoaching in achieving a DBP outcome [12,18,21].

People with DM who underwent the telecoaching had a lower diastolic blood pressure than the controls. A meta-analysis, as shown in Figure 8, assessed the intervention vs. the control efficacy of the telecoaching on the diastolic blood pressure with a *p* = 0.14 with a pooled MD of −0.87 (95% CI: −2.02–0.28). The telecoaching was found to not significantly decrease the DBP in people with DM. A heterogeneity was not found (I^2^ = 0%; *p* = 0.85).

#### 3.4.6. Meta-Analysis Outcome of Total Cholesterol

**Figure 9:**
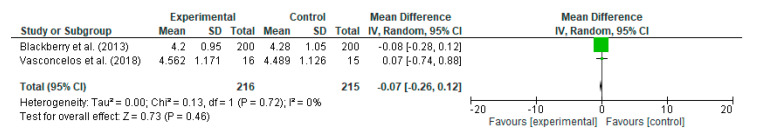
Forest plot intervention vs. control of telecoaching in achieving a total cholesterol outcome [12,15].

Regarding cholesterol, the people with DM who underwent the telecoaching had a lower cholesterol score than the controls. A meta-analysis, as shown in Figure 9, assessed the efficacy of the telecoaching on cholesterol with a *p* = 0.46 with a pooled MD of −0.07 (95% CI: −0.26–0.12). The telecoaching did not significantly decrease the total cholesterol level in people with DM. A heterogeneity was not found (I^2^ = 0%; *p* = 0.72).

#### 3.4.7. Meta-Analysis Outcome of Triglycerides

**Figure 10:**
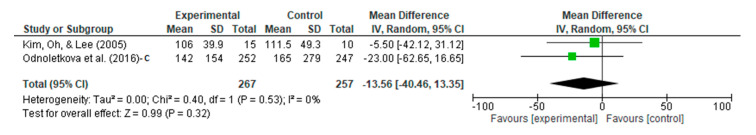
Forest plot intervention vs. control of telecoaching in achieving a triglycerides outcome [16,21].

In terms of triglycerides, the people with DM who underwent the telecoaching had lower triglycerides than the controls. A meta-analysis, as shown in Figure 10, assessed the efficacy of the telecoaching in lowering triglycerides with a *p* = 0.32 with a pooled MD of −13.56 (95% CI: −40.46–13.35). The telecoaching did not significantly decrease the triglyceride levels in people with DM. A heterogeneity was not found (I^2^ = 0%; *p* = 0.53).

#### 3.4.8. Meta-Analysis Outcome of HDL

**Figure 11:**
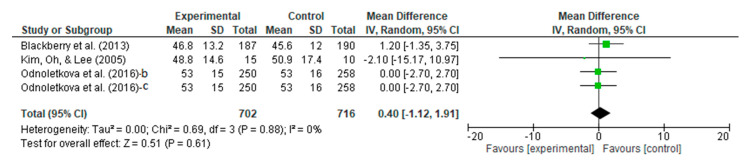
Forest plot intervention vs. control of telecoaching in achieving an HDL outcome [12,16,21,22].

Regarding the HDL, the people with DM who underwent the telecoaching had a higher HDL score than the controls. A meta-analysis, as shown in Figure 11, assessed the efficacy of the telecoaching on HDL with a *p* = 0.61 with a pooled MD of 0.40 (95% CI: −1.12–1.91). The telecoaching did not significantly increase the HDL-c levels in people with DM. A heterogeneity was not found (I^2^ = 0%; *p* = 0.88).

#### 3.4.9. Meta-Analysis Outcome of LDL

**Figure 12:**
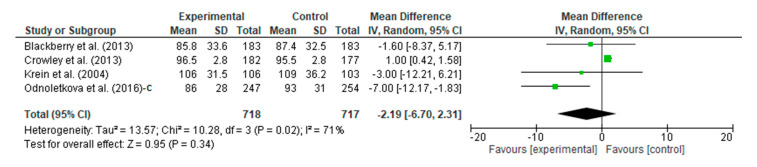
Forest plot intervention vs. control of telecoaching in achieving an LDL outcome [12,13,18,21].

A meta-analysis assessed the intervention vs. the control of the telecoaching on LDL. The results show, as in in Figure 12, a *p* = 0.34 with a pooled MD of −2.19 (95% CI: −6.70–2.31). The telecoaching did not significantly decrease the LDL-c levels in patients with DM. A heterogeneity was found (I^2^ = 71%, *p* = 0.02).

#### 3.4.10. Meta-Analysis Outcome of Weight

**Figure 13:**
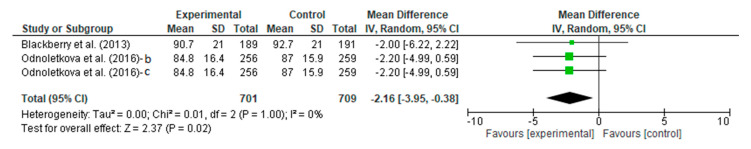
Forest plot intervention vs. control of telecoaching in achieving a weight outcome [12,21,22].

A meta-analysis assessed the intervention vs. the control efficacy of the telecoaching on weight. The result is shown in Figure 13, which depicts a significant effect with a *p* = 0.02 with a pooled MD of −2.16 kg (95% CI: −3.95–−0.38). The telecoaching was found to decrease weight in people with DM significantly. A heterogeneity was not found (I^2^ = 0%; *p* = 1).

#### 3.4.11. Meta-Analysis Outcome of BMI

**Figure 14:**
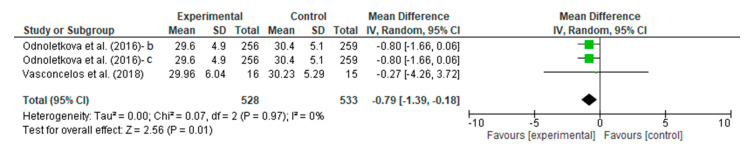
Forest plot intervention vs. control of telecoaching in achieving a BMI outcome [15,21,22].

The people with DM who underwent the telecoaching had a lower BMI score than the controls. A meta-analysis, as shown in Figure 14, assessed the efficacy of the telecoaching on lowering BMI scores significantly with a *p* = 0.01 with a pooled MD of −0.79 (95% CI: −1.39–−0.18). The telecoaching was found to decrease the BMI in people with DM significantly. A heterogeneity was not found (I^2^ = 0%; *p* = 0.97).

#### 3.4.12. Feasibility and Adherence to a Healthy Lifestyle

Telecoaching has been the focus of research to identify if it could help people adopt healthier habits. One randomized controlled trial study evaluated whether telecoaching effectively increased physical activity and diet. In addition, telecoaching was also observed to be feasible to be given to people as a self-care delivery model for diabetes mellitus. For example, a six-month follow-up study found that 19/21 persons completed it successfully, and that 98.5% were satisfied with the telecoaching program [14].

#### 3.4.13. Cost-Effectiveness Outcome

In terms of cost savings, telecoaching is considered effective in lowering costs. According to one study conducted in Belgium, telecoaching was relatively cost-effective [22].

## 4. Discussion

Based on the American Diabetes Association (ADA), the evaluations carried out in assessing diabetes mellitus can be seen from the levels of HbA1c and plasma glucose—both the fasting plasma glucose, 2 h plasma glucose, or random plasma glucose—and as a glycemic control [24]. Based on its pathophysiology, hyperglycemia can be caused by insulin resistance caused by excess fatty acids and proinflammatory cytokines, causing an inappropriate increase in glucagon [25]. Consequently, increased lipids and their components also play an essential role in evaluating diabetes mellitus.

Diabetes is one of the most widespread chronic diseases worldwide, impacting millions of people. It is also one of the major co-morbidities in COVID-19 infection deaths, second only to hypertension. In individuals with diabetes, the probability of hospital death with COVID-19 is 3.5 to 5 times higher than in non-diabetic individuals. Moreover, diabetes and other co-morbidities increase COVID-19-infected patients’ clinical state, increasing the probability of severe outcomes, including death [26]. Diabetes does not appear to aid COVID-19 infection, but obviously, this epidemic has altered the care of chronic diseases, such as diabetes mellitus, and the normal interactions between patients and nurses or physicians. During the COVID-19 pandemic, people with diabetes should not be left alone to handle their disease, and this has become a serious and urgent health issue [27]. Therefore, consultations conducted through telecoaching have proven to be very helpful in managing diabetes over the entirety of the COVID-19 pandemic and possibly for other transmissible outbreaks as well [28].

Telecoaching is a diagnosis or treatment delivered via personal communication and using technology, either through mobile phones, computers, or tablets [29]. The occurrence of a pandemic that requires people to minimize contact with each other makes telecoaching an innovative step in monitoring patients’ illnesses. The study by Robson et al. shows that among the various methods and types of telecoaching used, phone-based telecoaching has a superior effect compared to other studies, even though it can be difficult to judge what methods benefit patients the most [30]. Other studies have also shown a positive relationship between an increase in the frequency of telecoaching with decreasing HbA1C scores in patients with T2DM [31].

Our systematic review and meta-analysis are the first to assess various types of outcomes from the effect of telecoaching on people with T2DM. We assessed a meta-analysis of HbA1C scores, blood glucose levels, SBP, total cholesterol, triglycerides, HDL, LDL, weight, and BMI all at once. Our study shows that telecoaching significantly impacts the HbA1C scores, blood glucose levels, SBP, weight, and BMI. This telecoaching assessment method represents a strategic and potential step, especially with its implementation in line with the COVID-19 pandemic.

Research conducted by Odnoletkova et al. (2016) stated that in the follow-up carried out on people with Type 2 diabetes mellitus at the eighteenth week with an intervention in the form of nurse-led telecoaching, there was a difference in HbA1c with a significant value (−0.2 mmol/mol, and *p* = 0.046) in all persons and this was −0.4, and *p* < 0.023 in the elevated HbA1c subgroup. In addition, the intervention was also able to reduce the SBP and DBP with a reduction of 1.3% in the control group (*p* = 0.011) [23]. These results are consistent with other systematic reviews and meta-analyses with the same intervention in people with Type 2 diabetes mellitus that had significant results in the reduction of glycated hemoglobin (a pooled mean difference = −1.23, and *p* < 0.00) and SBP (a pooled mean difference = −2.22 and *p* < 0.01) [32]. However, the BMI, which can be observed as a linear comparison between hypertension and diabetes mellitus, was also assessed and obtained a significant result with a mean of −0.4 kg/m^2^ (*p* = 0.003) [33].

Another study with an intervention in the form of in-app coaching also assessed the BMI with a reduced but not statistically significant BMI result, where persons changed their medications during the research process. As for the other results in the form of A1c, which acts as glycemic control, significant results were obtained with a mean of −0.86% (*p* < 0.001) [7]. Clinical outcomes other than the BMI were assessed in another study with a significantly reduced total lipid by a mean of −6 mg/dl (*p* = 0.012) and a reduction in LDL of 8.9% (*p* = 0.011) in the control group [23].

The use of telecoaching can also improve the quality of life by a mean of +1.83 (*p* < 0.0001), followed by a decrease in depression. In another study conducted in Belgium, telecoaching was said to be cost-effective with ICERs of EUR 5569 and EUR 4615 [22]. All the studies concluded that telecoaching could be an effective form of self-care delivery in managing glycemic control and improving clinical outcomes, supported by cost-effectiveness and contributing to enhancing the quality of life by reducing depression.

Despite telecoaching showing potential as a delivery self-care model in DM, there is a challenge in implementing telecoaching that must be resolved. First, with technological advances raising the patient demand for mobile and remote healthcare services, national healthcare authorities must ensure their feasibility and quality requirements in chronic care, where their effectiveness has been shown [34,35]. A framework must be developed to support the necessary changes, including a conceptualization of patient education in multi-morbidities—including the qualification of the educator, the process of providing education and its evaluation, and the scope of interaction with a care team—and legal clarity on information security and privacy, professional liability, and remuneration for providers’ performances [36]. Second, documenting informed permission and protecting medical information are emphasized as being necessary to avoid ethical issues. Universities, training institutions, and scientific societies must provide integrated health training with telenursing as soon as practicable [37]. Finally, patient self-management support programs are severely underfunded at present, and economics-based health research, that is both necessary and relevant is vastly underrepresented as compared to reviews of drugs [22]; consequently, to better inform policymakers’ decisions about healthcare budget allocations, more economic analyses of programs are required.

We have acknowledged some limitations in this study. First, the included studies in this meta-analysis were mostly conducted in upper-middle-income countries and countries such as the United States, in Europe, and in East Asia; thus, this study’s results should not be generalized to another country with different economic, social, and cultural characteristics. Second, some included studies had a high bias due to a deviation from the intended intervention and missing outcome data. According to Hance, further study with a large sample size and robust methodology is needed to improve the quality of evidence. Third, this meta-analysis does not assess the moderating factors related to this study. Furthermore, a meta-regression is required to examine the moderating factors that potentially influenced the results. Fourth, although a dual independent review of the search results by two reviewers is generally recommended for systematic reviews, this approach is resource-intensive, which was our problem; therefore, this review process was conducted only by two reviewers and a two-step selection process. Finally, the lack of study during the COVID-19 pandemic has drastically limited the ability to trace the disease’s genuine management, which would only be possible based on historical information in normal conditions. This can only be accomplished in normal settings.

## 5. Conclusions

The systematic review and meta-analyses of the thirteen included studies for the meta-analysis found that nurse-led telecoaching with nurse telephone and mobile application models had significant effects on clinical outcomes, such as in HbA1c, systolic blood pressure, weight, and BMI. Moreover, the included RCT studies were conducted in a normal setting context. Consequently, this study only shows the potential interventions as a model to provide self-care for diabetes mellitus during COVID-19 and similar pandemics. Nevertheless, further studies are needed to evaluate the effects of telecoaching on other important variables and outcomes, such as examining a person’s experience, health outcomes, health equity, and the cost-effectiveness of the many emerging hybrid-care models. More economic studies of telecoaching programs are also needed to better inform policymakers’ decisions about the distribution of healthcare budgets. Additionally, a robust study of telecoaching’s efficacy in lower-middle-income countries is required.

## Figures and Tables

**Table 1:** Characteristics of Included Studies.

Study	Study Design	Location	Sample				Intervention			Control/Comparator	Follow-Up
			Characteristic	Size	Gender (Male %)	Age (Year)	Model	Frequency	Other Service		
Blackberry et al. (2013) [12]	RCT	Australia	People with Type 2 DM	440	57	62.8 ± 10.5	Telephone coaching by a practice nurse	Five telephone coaching sessions during six weeks.	N/A	Routine practice care	15 months
Crowley et al. (2013) [13]	RCT	USA	People with Type 2 DM	359	27.9	Intervention: 57 ± 12; Control: 56 ± 12	A telephone call from a nurse	Monthly telephone call for 12 months.	Electronic nurse communication facilitated the medication management.	Routine practice care	12 months
Kim and Oh (2003) [17]	RCT	South Korea	People with Type 2 DM	36	30.6	Intervention: 59.7 ± 7.3; Control: 60.9 ± 5.8	A telephone call by a nursing PhD student	Twice a week and weekly telephone call for 12 weeks.	Exercise medication adjustments and frequent blood glucose monitoring.	Routine practice care	3 months
Kim, Oh, and Lee (2005) [16]	RCT	South Korea	People with Type 2 DM	25	36	Intervention: 61.0 ± 6.1; Control: 60.4 ± 6.4	Telephone counseling by a doctoral nursing student	12 weeks	N/I	Routine practice care	3 months
Krein et al. (2004) [18]	RCT	USA	People with Type 2 DM	209	96.6	Intervention: 61.0 ± 10; Control: 61 ± 11	A telephone call from a nurse	According to individual patient needs.	Medication adjustment.	Usual care	18 months
Kumar et al. (2018) [7]	RCT	USA	People with DM	146	29	52.0 ± 9.0	Diabetes mobile app	12 weeks	N/A	Smartwatch (Apple Watch, Cupertino, CA)	3 months
Nesari et al. (2010) [19]	RCT	Iran	People with Type 2 DM	60	28.3	Intervention: 51.96 ± 7.61; Control: 51.3 ± 8.24	A telephone call by nurses	Twice a week and weekly telephone call for 12 weeks.	Medication adjustment based on glycemic level	Three-day education program	3 months
Odnoletkova et al. (2016)-c [21]	RCT	Belgium	People with Type 2 DM	574	61.5	N/I	N/I	N/I	N/A	N/I	N/I
Odnoletkova et al. (2016)-a [23]	RCT	Belgium	People with Type 2 DM	574	62	64	COACH program led by a nurse	Five telephone sessions of 30 min on average, spread over 6 months.	N/A	Routine practice care	18 months
Odnoletkova et al. (2016)-b [22]	RCT	Belgium	People with Type 2 DM	574	62	64	COACH program led by a nurse	five telephone sessions of a mean (range) duration of 30 (10–45) min, delivered at a mean (range) interval of 5 (3–8) weeks by a certified diabetes nurse educator (hereafter referred to as the ‘coach’) after a 5-day training course.	N/A	Routine practice care	18 months
Vasconcelos et al. (2018) [15]	RCT	Brazil	People with Type 2 DM	31	33.4	59.6	A program of guidance/coaching on the disease via telephone calls made by a researcher nurse	Twelve bi-weekly telephone contacts were made over a period of 24 weeks.	N/A	Routine practice care	6 months
Wayle et al. (2014) [14]	RCT	Canada	People with Type 2 DM	21	43	55.6 ± 12.3	NexJ Health Coach App	24 weeks	N/A	-	6 months
Weinberger et al. (1995) [20]	RCT	USA	People with Type 2 DM	251	98.9	Intervention: 63.2 ± 8.3; Control: 63.9 ± 8.6	Telephone education by nurses	Monthly telephone call for 12 months.	Evaluating prescribed regimens and emphasizing compliance, health monitoring, facilitate primary care.	N/I	12 months

Note. DM: Diabetes Mellitus; N/I: no information; N/A: not available; RCT: randomized controlled trial.

## Data Availability

More data is available in the author. Please contact the author for more data.

## References

[1] World Health Organization (2020). COVID 19 Public Health Emergency of International Concern (PHEIC) Global Research and Innovation Forum: Towards a Research Roadmap. Glob. Res. Collab. Infect. Dis. Prep..

[2] CDC People with Certain Medical Conditions. https://www.cdc.gov/coronavirus/2019-ncov/need-extra-precautions/people-with-medical-conditions.html.

[3] Boulton A. (2021). Why access to diabetes care must not be another victim of the COVID-19 pandemic. Diabetes Res. Clin. Pract..

[4] Schulman-Green D., Jaser S.S., Park C., Whittemore R. (2016). A Metasynthesis of Factors Affecting Self-Management of Chronic Illness. J. Adv. Nurs..

[5] Shi C., Zhu H., Liu J., Zhou J., Tang W. (2020). Barriers to self-management of type 2 diabetes during COVID-19 medical isolation: A qualitative study. Diabetes Metab. Syndr. Obes. Targets Ther..

[6] Yancy C.W., Jessup M., Bozkurt B., Butler J., Casey D.E., Drazner M.H., Fonarow G.C., Geraci S.A., Horwich T., Januzzi J.L. (2013). 2013 ACCF/AHA guideline for the management of heart failure: Executive summary: A report of the American college of cardiology foundation/american heart association task force on practice guidelines. J. Am. Coll. Cardiol..

[7] Kumar S., Moseson H., Uppal J., Juusola J.L. (2018). A Diabetes Mobile App With In-App Coaching From a Certified Diabetes Educator Reduces A1C for Individuals With Type 2 Diabetes. Diabetes Educ..

[8] Page M.J., McKenzie J.E., Bossuyt P.M., Boutron I., Hoffmann T.C., Mulrow C.D., Shamseer L., Tetzlaff J.M., Akl E.A., Brennan S.E. (2021). The PRISMA 2020 statement: An updated guideline for reporting systematic reviews. BMJ.

[9] Riley R.D., Moons K.G.M., Snell K.I.E., Ensor J., Hooft L., Altman D.G., Hayden J., Collins G.S., Debray T.P.A. (2019). A guide to systematic review and meta-analysis of prognostic factor studies. BMJ.

[10] Higgins J.P.T., Thompson S.G., Deeks J.J., Altman D.G. (2003). Measuring inconsistency in meta-analyses. BMJ.

[11] Duval S., Tweedie R. (2000). Trim and fill: A simple funnel-plot-based method of testing and adjusting for publication bias in meta-analysis. Biometrics.

[12] Blackberry I.D., Furler J.S., Best J.D., Chondros P., Vale M., Walker C., Dunning T., Segal L., Dunbar J., Audehm R. (2013). Effectiveness of general practice based, practice nurse led telephone coaching on glycaemic control of type 2 diabetes: The Patient Engagement And Coaching for Health (PEACH) pragmatic cluster randomised controlled trial. BMJ.

[13] Crowley M.J., Powers B.J., Olsen M.K., Grubber J.M., Koropchak C., Rose C.M., Gentry P., Bowlby L., Trujillo G., Maciejewski M.L. (2013). The cholesterol, hypertension, and glucose education (CHANGE) study: Results from a randomized controlled trial in African Americans with diabetes. Am. Heart J..

[14] Wayne N., Ritvo P. (2014). Smartphone-Enabled Health Coach Intervention for People with Diabetes from a Modest Socioeconomic Strata Community: Single-Arm Longitudinal Feasibility Study. J. Med. Internet Res..

[15] Vasconcelos H.C.A.d., Neto J.C.G.L., Araújo M.F.M.d., Carvalho G.C.N., de Souza Teixeira C.R., Freitas R.W.J.F.d., Damasceno M.M.C. (2018). Telecoaching programme for type 2 diabetes control: A randomised clinical trial. Br. J. Nurs..

[16] Kim H.-S., Oh J.-A., Lee H.-O. (2005). Effects of Nurse-Coordinated Intervention on Patients with Type 2 Diabetes in Korea. J. Nurs. Care Qual..

[17] Kim H.-S., Oh J.-A. (2003). Adherence to Diabetes Control Recommendations: Impact of Nurse Telephone Calls. J. Adv. Nurs..

[18] Krein S.L., Klamerus M.L., Vijan S., Lee J.L., Fitzgerald J.T., Pawlow A., Reeves P., Hayward R.A. (2004). Case management for patients with poorly controlled diabetes: A randomized trial. Am. J. Med..

[19] Nesari M., Zakerimoghadam M., Rajab A., Bassampour S., Faghihzadeh S. (2010). Effect of telephone follow-up on adherence to a diabetes therapeutic regimen. Jpn. J. Nurs. Sci..

[20] Weinberger M., Kirkman M.S., Samsa G.P., Shortliffe E.A., Landsman P.B., Cowper P.A., Simel D.L., Feussner J.R. (1995). A nurse-coordinated intervention for primary care patients with non-insulin-dependent diabetes mellitus: Impact on glycemic control and health-related quality of life. J. Gen. Intern. Med..

[21] Odnoletkova I., Buysse H., Nobels F., Goderis G., Aertgeerts B., Annemans L., Ramaekers D. (2016). Patient and provider acceptance of telecoaching in type 2 diabetes: A mixed-method study embedded in a randomised clinical trial. BMC Med. Inform. Decis. Mak..

[22] Odnoletkova I., Ramaekers D., Nobels F., Goderis G., Aertgeerts B., Annemans L. (2016). Delivering Diabetes Education through Nurse-Led Telecoaching. Cost-Effectiveness Analysis. PLoS ONE.

[23] Odnoletkova I., Goderis G., Nobels F., Fieuws S., Aertgeerts B., Annemans L., Ramaekers D. (2016). Optimizing diabetes control in people with Type 2 diabetes through nurse-led telecoaching. Diabet. Med..

[24] American Diabetes Association (2010). Diagnosis and Classification of Diabetes Mellitus. Diabetes Care.

[25] Sapra A., Bhandari P. (2022). Diabetes Mellitus.

[26] Mantovani A., Byrne C.D., Zheng M.-H., Targher G. (2020). Diabetes as a risk factor for greater COVID-19 severity and in-hospital death: A meta-analysis of observational studies. Nutr. Metab. Cardiovasc. Dis..

[27] Kreutzenberg S.V.d. (2022). Telemedicine for the Clinical Management of Diabetes; Implications and Considerations After COVID-19 Experience. High Blood Press. Cardiovasc. Prev..

[28] Nørgaard K. (2020). Telemedicine Consultations and Diabetes Technology During COVID-19. J. Diabetes Sci. Technol..

[29] CSU MarComm Staff (2016). Telecoaching Emerges as Tool in Nutrition and Medicine.

[30] Robson N., Hosseinzadeh H. (2021). Impact of Telehealth Care among Adults Living with Type 2 Diabetes in Primary Care: A Systematic Review and Meta-Analysis of Randomised Controlled Trials. Int. J. Environ. Res. Public Health.

[31] Pimouguet C., Le Goff M., Thiébaut R., Dartigues J.F., Helmer C. (2011). Effectiveness of disease-management programs for improving diabetes care: A meta-analysis. Can. Med. Assoc. J..

[32] Chen D.Y.-M., Wu X.V., Chan E.Y., Goh Y.S. (2019). Nurse-Led Tele-Coaching on Modifiable Cardiovascular Risk Factors in People with Type 2 Diabetes Mellitus: A Systematic Review and Meta-Analysis. Worldviews Evid. Based Nurs..

[33] Bays H.E., Chapman R.H., Grandy S., The SHIELD Investigators’ Group (2007). The relationship of body mass index to diabetes mellitus, hypertension and dyslipidaemia: Comparison of data from two national surveys. Int. J. Clin. Pract..

[34] Komariah M., Maulana S., Platini H., Pahria T. (2021). A Scoping Review of Telenursing’s Potential as a Nursing Care Delivery Model in Lung Cancer During the COVID-19 Pandemic. J. Multidiscip. Healthc..

[35] Maulana S., Trisyani Y., Mirwanti R., Amirah S., Kohar K., Priyatmoko Putri A.I., Novianti E. (2022). The Potential of Cardiac Telerehabilitation as Delivery Rehabilitation Care Model in Heart Failure during COVID-19 and Transmissible Disease Outbreak: A Systematic Scoping Review of the Latest RCTs. Medicina.

[36] Lee J., Rho M.J. (2013). Perception of Influencing Factors on Acceptance of Mobile Health Monitoring Service: A Comparison between Users and Non-users. Healthc. Inform. Res..

[37] Nittari G., Khuman R., Baldoni S., Pallotta G., Battineni G., Sirignano A., Amenta F., Ricci G. (2020). Telemedicine Practice: Review of the Current Ethical and Legal Challenges. Telemed. e-Health.

